# Pathological alteration and therapeutic implications of sepsis-induced immune cell apoptosis

**DOI:** 10.1038/s41419-019-2015-1

**Published:** 2019-10-14

**Authors:** Chao Cao, Muming Yu, Yanfen Chai

**Affiliations:** 1 Tianjin Medical University General Hospital, Tianjin, China; 2 Tianjin Medical University, Tianjin, China; 3 Department of Internal Medicine, The University of Iowa Carver College of Medicine, Iowa City, IA USA

**Keywords:** Immunological disorders, Infectious diseases, Immunopathogenesis

## Abstract

Sepsis is a life-threatening organ dysfunction syndrome caused by dysregulated host response to infection that leads to uncontrolled inflammatory response followed by immunosuppression. However, despite the high mortality rate, no specific treatment modality or drugs with high efficacy is available for sepsis to date. Although improved treatment strategies have increased the survival rate during the initial state of excessive inflammatory response, recent trends in sepsis show that mortality occurs at a period of continuous immunosuppressive state in which patients succumb to secondary infections within a few weeks or months due to post-sepsis “immune paralysis.” Immune cell alteration induced by uncontrolled apoptosis has been considered a major cause of significant immunosuppression. Particularly, apoptosis of lymphocytes, including innate immune cells and adaptive immune cells, is associated with a higher risk of secondary infections and poor outcomes. Multiple postmortem studies have confirmed that sepsis-induced immune cell apoptosis occurs in all age groups, including neonates, pediatric, and adult patients, and it is considered to be a primary contributing factor to the immunosuppressive pathophysiology of sepsis. Therapeutic perspectives targeting apoptosis through various strategies could improve survival in sepsis. In this review article, we will focus on describing the major apoptosis process of immune cells with respect to physiologic and molecular mechanisms. Further, advances in apoptosis-targeted treatment modalities for sepsis will also be discussed.

## Facts

- Sepsis leads to uncontrolled inflammatory response with immunosuppression.
- Immune cell alteration induced by uncontrolled apoptosis is a major cause of profound immunosuppression.
- Therapeutic perspectives targeting apoptosis through various strategies could improve survival in sepsis.

## Open questions

- It is known that apoptotic depletion of immune cells is responsible for immune dysfunction; however, how do immune cells, including innate and adaptive immune cells, change in the course of sepsis?
- What are the specific molecular mechanisms and interactions involved in the pathways mediating physiological alteration of immune cells?
- Are there specific pathways and related factors that can be diagnostic and therapeutic targets for sepsis-induced immunosuppression?

## Introduction

Sepsis is a major public health challenge worldwide owing to protracted inflammation, immune suppression, susceptibility to infections, and even death^1^. Despite improvements in the understanding of the pathophysiology of sepsis and therapeutic strategies, no specific therapeutic agent for the treatment of sepsis has been approved to date^2,3^.

Sepsis treatment, including antibiotic therapies, ventilator management, blood glucose maintenance, and resuscitative strategies, has rapidly progressed^4,5^, particularly the supportive therapies recommended by the Surviving Sepsis Campaign^6^. However, few novel effective therapies have been identified, and the incidence of sepsis has increased^7,8^, with approximately 31.5 million cases of sepsis, 19.4 million severe sepsis, and 5.3 million deaths reported annually^9^. Sepsis with subsequent multiple organ dysfunction remains the leading cause of mortality in hospitalized patients, and it is projected to increase at an alarming rate over the next two decades^10^. Even more alarming is the increasing rate of sepsis-associated intensive care unit (ICU) mortality, which is the most common cause of death in ICU patients; severe sepsis accounts for >50% of ICU mortality^11^. Patients with sepsis are also hospitalized longer and have an eight times higher risk of death during hospitalization than other inpatients^12^.

**Fig. 1:**
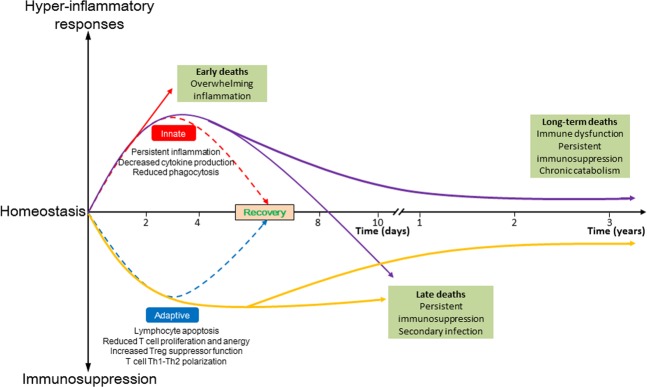
Immune response in sepsis. Early activation of both innate and adaptive immune response is involved in the pathogenesis of sepsis. The peak mortality rates during the early period (top red line) were due to overwhelming inflammatory response, also known as “cytokine storm,” which comprises fever, refractory shock, inadequate resuscitation, and cardiac or pulmonary failure. Meanwhile, mortality at the later period is due to persistent immunosuppression with secondary infections that results in organ injury and/or failure. Although more sophisticated ICU care has improved mortality, patients still die at the later period or after several years owing to the persistent immunosuppression, immune dysfunction, or chronic catabolism

**Fig. 2:**
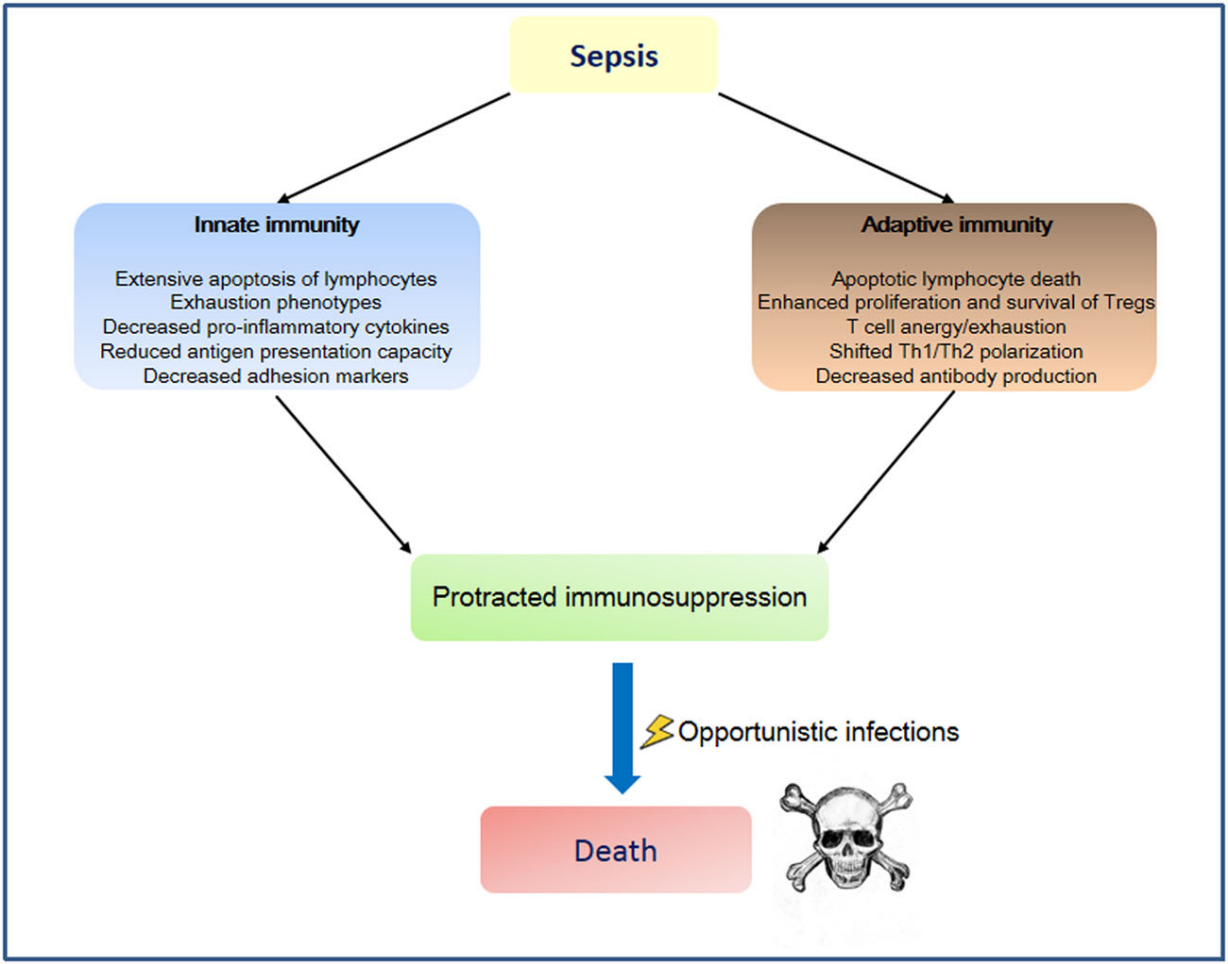
Alterations in innate and adaptive immunity in the pathophysiology of sepsis. Early activation of innate immunity is the first line of defense against infection and plays a central role in the initiation of adaptive immunity. However, in sepsis, excessive immune responses lead to several alterations in innate and adaptive immunity that contribute to protracted immunosuppression and increase the risk for opportunistic infection. In some way, sepsis can be considered as a race to the death between host immune response and pathogens that seek an advantage by impairing the host immune defenses

In general, the pathophysiology of sepsis is considered as an initial hyperinflammatory phase that lasts for several days followed by a more protracted immunosuppressive phase^13^. The current death distribution indicates peaks during the early phase, although at a lower magnitude, and another peak after 2–3 months that continues to increase over the next 3 years^14,15^ (Fig. 1). However, varying definitions and ineffective clinical strategies have led to discrepancies in the incidence and mortality rates of sepsis^16^, and thus the criterion should be redefined^17^. In the third International Consensus for Sepsis and Septic Shock, sepsis was clinically defined as a life-threatening condition of organ dysfunction caused by a dysregulated immune response to infection^18^. These changes shift the focus of pathophysiology on sepsis-induced immune dysfunction and long-term outcomes of sepsis patients who survive from a fatal stage owing to sophisticated care in the ICU^19^. Sepsis can be considered as a race to death between the pathogens and host immune response, where the pathogens seek an advantage by incapacitating various aspects of host immunity^20^. Numerous studies have shown that a large number of patients who died of sepsis had unresolved opportunistic infections^21,22^ and immunosuppressive features^10,23,24^ (Fig. 2). Several mechanisms are responsible for sepsis-induced immunosuppression, including apoptotic depletion of immune cells, increased expression of negative costimulatory molecules, increased regulatory T (Treg) cell expression, expression of programmed cell death (PD)-1 on CD4^+^ T cells, and cellular exhaustion^23^. Of these, immune cell apoptosis has been increasingly recognized as a key factor in the pathophysiology of septic complications^25–27^.

Widespread lymphocyte apoptosis occurring in the lymphoid (spleen, thymus, and lymph nodes) and other organs^28^ that results in impaired immune cell activity (including that of neutrophils, monocyte and macrophages, B cells, natural killer cells [NK cells], and dendritic cells [DCs]) is a crucial contributing factor to the development of the immunosuppressive phase of sepsis^29^. Multiple studies have reported that preventing immune cell apoptosis could markedly improve survival^10,30,31^. In this review, we will highlight the importance of sepsis-induced immune cell apoptosis, including the role of innate and adaptive immune cells in the pathogenesis of sepsis, alterations in their immune metabolic stage, and the clinical implications and potential therapeutic interventions.

## Apoptosis in sepsis

Apoptosis is a tightly regulated form of cell death that is vital in both embryo implantation and development and turnover of tissues during maturation^32^. During sepsis-induced immunosuppression, apoptosis plays a pivotal role in the selection of immune cell populations and maintenance of functional immune responses^33^. Cell death in innate and adaptive immune systems benefits the host by downregulating inflammatory response in sepsis, but the extensive loss of immune cells may compromise the ability of the host to eliminate invading pathogens. Immune cell apoptosis in lymphoid tissues and gut-associated lymphoid tissues could cause marked depletion of immune cells, including monocytes and macrophages, DCs, NK cells, and B cells^34^, contributing to immune suppression or secondary infection^35^. The depletion of these immune cells is a universal finding in all age groups and is particularly noteworthy because it occurs during life-threatening infection when clonal expansion of lymphocytes should be occurring. Although circulating lymphocytes undergo significant apoptosis, no apparent apoptosis in the heart, kidneys, lungs, and other substantive organs occurs during the progression of sepsis^36^. Sepsis-induced apoptosis of immune cells could undermine host immunity through anergy, latent infection reactivation, and susceptibility to secondary infections^37^. Importantly, the magnitude of lymphocyte apoptosis is possibly valuable for determining the severity of sepsis. However, the activity of immune cells differs under diverse apoptotic signals during sepsis. Cellular death through apoptosis directly leads to microvascular dysfunction and organ failure during sepsis, with apoptotic immune cells contributing to secondary infection or immune suppression.

## Mechanisms and consequences of apoptosis in sepsis

Although immune cell depletion is a crucial event in the pathology of sepsis-induced immunosuppression, the mechanisms responsible for this are not fully understood^38^. One theory is that sepsis could affect apoptosis-induced decrease in the number of DCs, which are the most potent antigen-presenting cells (APCs). This then leads to impaired innate and adaptive immune response^25^. In addition, the profound decrease in the number of some critical cells could influence adaptive immune responses between the innate and adaptive systems. The other theory is that some factors, including steroids, cytokines (tumor necrosis factor [TNF]-α, high mobility group box-1 protein, FasL, and heat shock protein), could regulate apoptosis by directly modulating the activities of caspase-8 in the death-induced signaling complex or by changing the levels of death and survival factors that control the Fas apoptotic pathway. Conversely, the release of anti-inflammatory cytokines, such as interleukin (IL)-10 and transforming growth factor beta, could accelerate apoptosis.

This process ultimately leads to major consequences. First, with respect to immune response, excessive apoptosis causes massive loss of immune cells. For example, the depletion of macrophages and NK cells impairs microorganism clearance^39,40^, which leads to protracted inflammatory responses. The second major consequence is that uncontrolled apoptosis of immune cells results in immunological tolerance with anti-inflammatory properties^41^. During apoptosis, the release of pro-inflammatory cytokines is inhibited, but the secretion of anti-inflammatory factors is activated, indicating a shift from T helper type 1 (Th1) to Th2 cytokine production^25,42^. The sepsis-induced functional and quantitative changes in immune cells result in lymphopenia, with progression of immune paralysis.

## Apoptosis-induced lymphopenia during sepsis

Sepsis could affect the function of virtually all types of immune cells. In the following section, we will discuss these various immunosuppressive effects on the different cells of the innate and adaptive immune systems.

### Neutrophils

Neutrophils are produced in the bone marrow (BM) and released into the circulation as the most prevalent and integral innate cell population. They are fundamental components of innate immunity and are essential for microbial eradication and for sepsis survival^43^. Neutrophils are the most abundant leukocyte in systemic circulation. They are also present in small amounts in the spleen, liver, and lung^44,45^ apart from the BM, and thus they are critical for early immune response^46,47^. In addition, neutrophils may function as APCs and mediate between innate and adaptive responses in various pathological infections^48^.

**Fig. 3:**
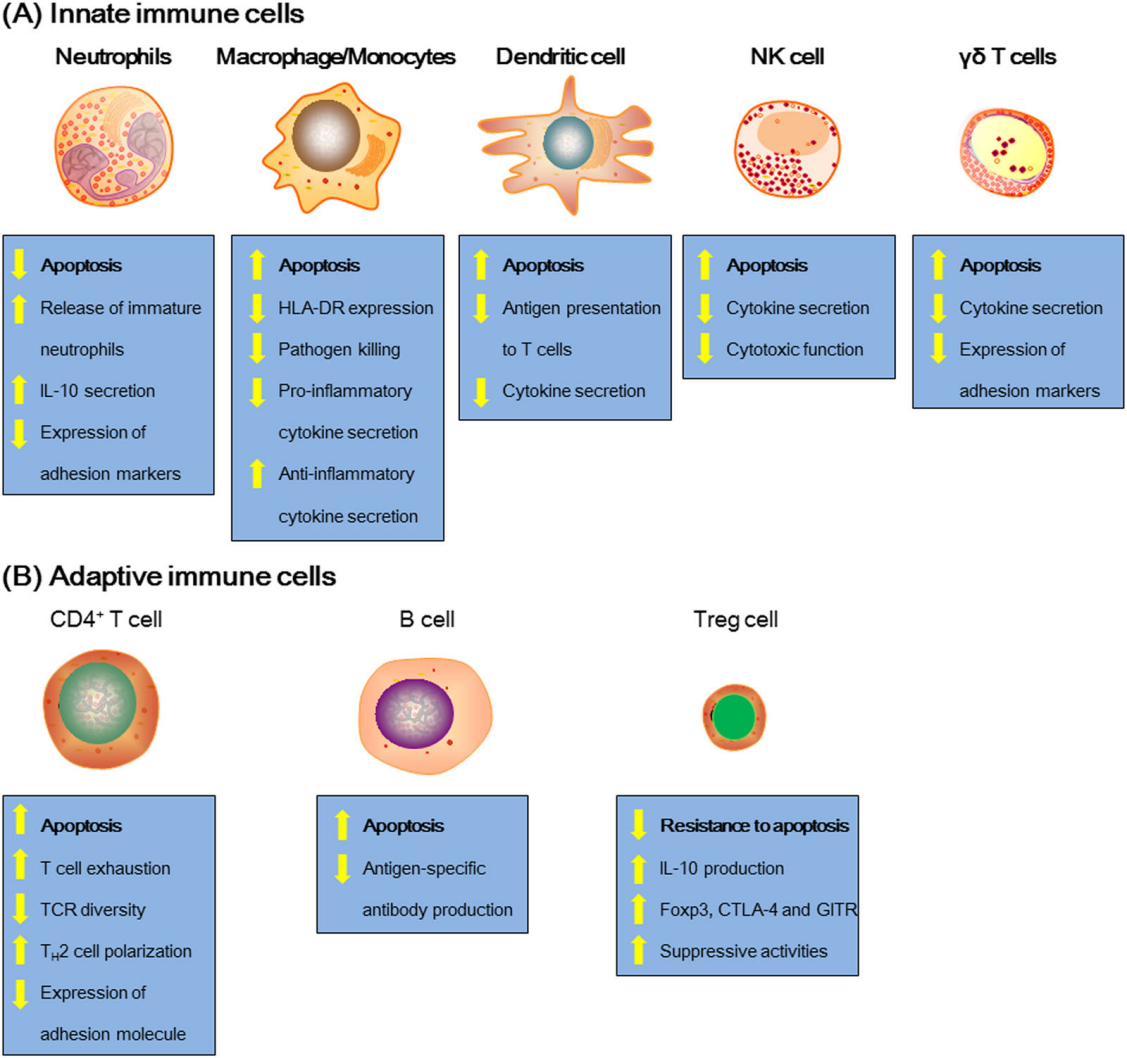
Sepsis alters innate and adaptive immune cells. Sepsis-induced immune paralysis is characterized by immunological defects that impair host immunity. Lymphoid cell loss, often resulting in the diminished capacity to fight and eliminate pathogens, is a primary feature of immune suppression during sepsis. Altered immune cell function induced by uncontrolled apoptosis is a major cause of profound immunosuppression. Lymphocyte apoptosis, including that of innate immune cells and adaptive immune cells, is associated with a higher risk of secondary infections and poor outcome in various diseases. As shown here, sepsis rapidly triggers profound apoptosis in macrophages/monocytes, dendritic cells, NK cells, γδ T cells, CD4^+^ T cells, and B cells. However, apoptosis of neutrophils is delayed, and Treg cells are more resistant to sepsis-induced apoptosis. Immune cell depletion due to apoptosis is the primary mechanism of sepsis-induced immune suppression

**Fig. 4:**
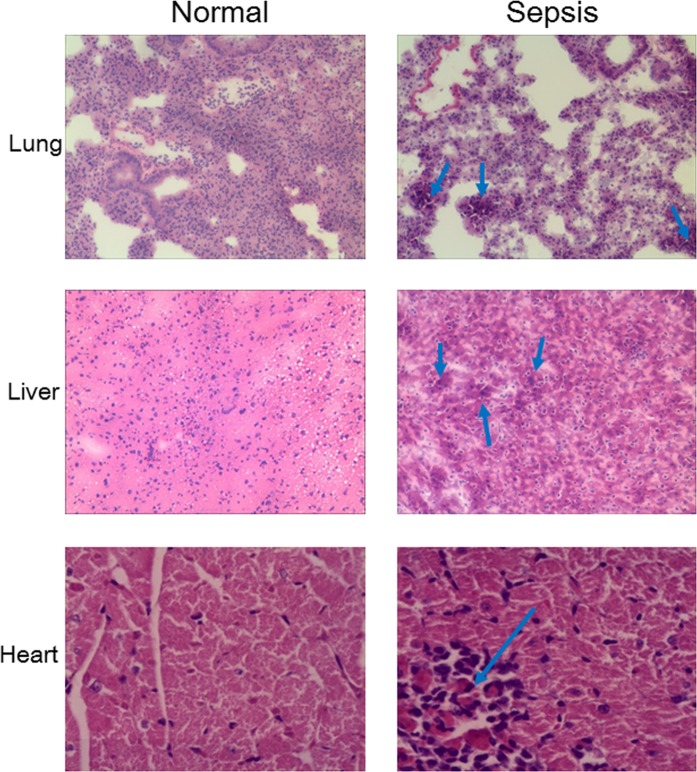
Sepsis-induced delayed apoptosis and recruitment of neutrophils into tissues lead to multiple organ dysfunction syndrome. The apoptosis of neutrophils is delayed during the first 24 h after the initiation of sepsis. Then neutrophils are recruited and infiltrate into tissues, aggravating the ongoing neutrophil dysfunction with persistent immune dysfunction and inflammation persistence. Neutrophil infiltration in the lungs is a pathological hallmark of sepsis-induced acute lung injury or acute respiratory distress syndrome as well as of organ dysfunction in the liver and heart. Representative histological changes in tissues are shown in hematoxylin and eosin-stained samples (original magnification ×400). Arrows denote the recruitment and infiltration of neutrophils into tissues

In physiological conditions, neutrophils are constitutively pro-apoptotic^49^ and are short-lived granulocytes that undergo energy- and caspase-dependent apoptosis within 24 h^46^. However, in the early stage of sepsis, the level of neutrophils increase rapidly owing to delayed neutrophil apoptosis^25^, as evidenced by the high neutrophil count observed in animals and sepsis patients only during the first 24 h of sepsis initiation^50^. This then leads to persistent neutrophil dysfunction, compounded by the release of immature neutrophils from the BM that culminates in neutrophil deficits in oxidative burst^51^, cell migration^52,53^, complement activation, and bacterial clearance^54^, all of which contribute to immune dysfunction and persistent inflammation (Fig. 3). In addition, immature neutrophils are produced and released from the BM, and their circulation aggravates the delayed apoptosis^25^. Thus the inhibition of neutrophil apoptosis in sepsis could be harmful to host immunity, and neutrophil infiltration into tissues could damage organ function^23,55^. For example, neutrophil infiltration in the lungs is a pathological hallmark of sepsis-induced acute lung injury or acute respiratory distress syndrome^56^. Increased circulating neutrophils result in a dysregulated immune response by releasing cytokines and reactive oxygen species at sites distal to the infectious focus, leading to multiple organ failure (Fig. 4). However, accelerated apoptosis resulted in inflammation resolution in several preclinical models of injury^57,58^.

Various mechanisms governing neutrophil apoptosis have been implicated in sepsis. Intravenous lipopolysaccharide or endotoxin could increase the levels of various gene transcripts in neutrophils, which result in the suppression of neutrophil apoptosis^59^. The delayed apoptosis of neutrophils is associated with disease severity^60^, whereas increased apoptosis is beneficial for fighting infection in hemorrhagic shock^61^. Meanwhile, delayed neutrophil apoptosis but accelerated apoptosis of other immune cells might impair host immune system by increasing the dephosphorylation of epithelial cell caspase-8^62^. Although delayed neutrophil apoptosis results in increased neutrophil counts, some functional deficit in neutrophils are evident during the early and late time points in sepsis patients. Further, some functions decline with protracted sepsis^63^. More interestingly, neutrophil deficiency hastens the development of nosocomial and secondary infections^64^, which is probably due to impaired bacterial clearance and altered pulmonary cytokine response^65^. Currently, neutrophil apoptosis-induced lymphopenia is given more attention for its involvement in the development of secondary infection and as a potential predictor of mortality at 1 year after sepsis^66^. One potential mechanism by which apoptosis-induced lymphopenia occurs is that some neutrophil subsets during sepsis could secrete excessive amounts of IL-10, which restrains the proliferation of T lymphocyte^67^. Another mechanism is that complex interactions between the neutrophils and complement system could also cause complement-induced innate immune damage during sepsis^68^. In addition, von Gunten et al. reported that the apoptosis response of neutrophils to a death stimulus (Siglec-9 cross-linking) varies significantly between patients and at different stages of septic shock^69^. The feasibility of neutrophil function and immature neutrophil count as predictors of sepsis has also been evaluated based on the delayed neutrophil apoptosis observed in septic animals and patients^70^.

### Monocytes and macrophages

The impact of sepsis on monocyte subpopulations has been extensively studied. The most notable change during sepsis is that the impairment of bold monocytes from the patients initiates “endotoxin tolerance” and leads to poor outcome. Monocytes and macrophages are major components of the innate immune system and play pivotal roles in orchestrating host immune response during sepsis^71^. At the early phase of sepsis, two different lymphocytes secrete an increased level of pro-inflammatory factors and chemokines, which aggravate the inflammatory response^72,73^ and could contribute to increased mortality rate^74^. As sepsis progresses, the excessive apoptosis of monocytes and macrophages could result in immunosuppression and the higher risk of secondary infection or mortality^22,75^. Furthermore, monocyte and macrophage dysfunction leads to decreased release of pro-inflammatory cytokines, such as TNF-α, IL-1β, IL-6, and IL-12, whereas the release of anti-inflammatory mediators, such as IL-1 receptor antagonist (IL-1ra) and IL-10, is neither impaired nor enhanced (Fig. 3)^76–78^. These changes indicate that intracellular signaling has shifted from production of pro-inflammatory cytokines toward anti-inflammatory mediators associated with nosocomial infections and increased mortality^14^.

Aside from accelerated apoptosis during sepsis, functional defects in monocytes also contribute to the pathophysiology of sepsis-induced immunosuppression, which is characterized by suppressed mononuclear cell HLA-DR expression in monocytes^79^. The decreased level of monocyte HLA-DR could be considered as a marker of monocyte anergy^80^, as it impairs the function of monocytes^81^ and decreases lymphocyte proliferation in response to invading pathogens^82^. Notably, HLA-DR expression is a reliable predictor for the development of nosocomial infections and mortality during sepsis^83–86^, and immunotherapies targeting monocyte function in patients with sepsis are being developed^87,88^.

Macrophages are essential in maintaining and activating host inflammatory responses^89^. Traditionally, macrophages perform two most well-known polarizations: M1 (i.e., expression of inflammatory cytokines) and M2 (i.e., expression of factors that resolve or suppress inflammatory responses)^90^. Macrophage phagocytosis leads to an imbalance of pro- and anti-inflammatory cytokines, resulting in polarization of macrophages to an M2 phenotype. This is characterized by predominant release of anti-inflammatory cytokines, such as IL-10 and IL-1ra, and decrease of pro-inflammatory cytokines^91^. Collectively, sepsis-induced extensive depletion of macrophages could seriously impair host antimicrobial defenses, but anti-apoptotic therapies targeting macrophages through anti-apoptotic proteins, such as modulation of Bcl-2 family members, could effectively ameliorate the host response and decrease sepsis-induced morbidity and mortality^92^.

### Dendritic cells

DCs arise from BM progenitors and seed the peripheral tissues as immature cells. They are the most potent APCs and play an essential role in pathogen recognition, regulation of immune response, and inflammation^93,94^ by linking the innate and adaptive immunity^95,96^. DCs are classified into two types: conventional DCs (cDCs) and plasmacytoid DCs (pDCs)^97^. cDCs, such as monocytes, can also secrete IL-12, while pDCs are similar to plasma cells and secrete large amounts of interferon (IFN)-α. DCs, including plasmacytoid and myeloid DCs, are markedly reduced in the spleen and circulation during sepsis^98^. Notably, the loss of DCs seems associated with worse clinical outcomes in sepsis including death and nosocomial infections^99–101^. For example, the number of DCs was markedly reduced in both the spleen and mesenteric nodes at 12 h in septic models created via cecal ligation puncture^102^. Although the excessive depletion of DCs could induce accelerated differentiation of monocytes into DCs^103,104^, it does not compensate for the excessive depletion of DCs, and the number of DCs continue to decrease along with impaired functional capability^105^. The levels of surface molecules associated with the function of DCs, including CD40 and CD86 as well as major histocompatibility complex (MHC)-DR, markedly decrease, but the release of IL-10 tends to increase (Fig. 3)^96,106^. These changes lead surviving DCs to transform to tolerogenic DCs, which are DCs that are unable to induce an allogeneic T cell activation but lead to either T cell anergy or Treg cell proliferation^107^. Under this condition, DCs with immunosuppressive properties that undermine immune responses are lost, thus leading to failure of activation of the immune response of effector T cells and ultimately results to an immunosuppressive state and organ injury^105,108^. Thus the profound depletion of DCs has been considered as an early predictive biomarker for the outcome of sepsis^109^.

There are several mechanisms involved in the induction of DC apoptosis, including abnormal activation of the nuclear factor of activated T cells (NFAT) and an influx of extracellular Ca^2+^ and calcineurin-dependent nuclear NFAT translocation^110^. Further investigation found that DCs profoundly affected the systemic impact of severe sepsis through Toll-like receptor (TLR) signaling pathways by increasing the expression of MHC class II antigen and costimulatory molecules CD80 and CD8^63^. Multiple reports have shown that blocking sepsis-induced DC apoptosis or augmenting DC function could enhance sepsis survival^111–113^, indicating that preventing DC apoptosis could be a promising treatment strategy for sepsis.

### NK cells

NK cells have been heavily studied in sepsis due to their low number in the circulation but high numbers in tissues^114^; thus their contribution to the pathophysiology of sepsis remains unclear^115^. Only 5–15% of blood lymphocytes do not express specific receptors^116^. Traditionally, NK cells are recognized as immune regulators and are divided into different subpopulations based on CD16 and CD56 expression^117^. In humans, NK cells are characterized as CD3^−^ NKp46^+^ CD56^+^ cells^118^. Under normal conditions, NK cells can induce a rapid, non-specific innate immune response against intracellular bacteria, pyogenic bacteria, fungi, protozoa^119^, and viral infections^120,121^ through direct cytotoxicity against virus-infected cells and early production of cytokines that can inhibit viral replication. Further, NK cells are the major producers of IFN-γ^122^. In addition, NK cells play an important role in initiating host defense and coordinating innate and adaptive immune responses during sepsis^123^.

The most important change is that the accelerated apoptosis of NK cells leads to the decreased number of circulating NK cells^124,125^ that lasts for several weeks during sepsis^126^. Both CD56^hi^ and CD56^lo^ NK cell subpopulations are altered, and this is associated with increased mortality in patients with sepsis^127,128^. More importantly, the cytotoxic function of NK cell is decreased^40^, which contributes to sepsis-induced immunosuppression (Fig. 3). Furthermore, the decreased level of IFN-γ caused by excessive apoptosis of NK cells increases the risk of secondary infection^129^.

The loss of NK cells directly affects the immune responses in sepsis patients and thus may be a potential target for therapeutic intervention^130,131^. For example, PD-1/programmed cell death ligand-1 (PD-L1) blockade-based immunotherapy can be used during sepsis-associated immunosuppression that develops due to the loss of protective function of NK cells^132,133^. Thus NK cell-based immunotherapy and immunomodulatory molecules specifically targeting NK cells and their immunometabolism may open a future avenue to target sepsis^134^.

### Gamma delta (γδ) T cells

γδ T cells are a distinct lymphocyte population with a unique and expansive function. They play a pivotal role in protecting tissues against bacterial, viral, and parasitic pathogen damage^135^. γδ T cells comprise a small subset of T cells that possess a distinct T cell receptor (TCR) on their cell surface, that is, a TCR comprised of one γ chain and one δ chain. These cells exhibit features of both innate and adaptive immunity and play an indispensable role in host defense, immune surveillance, and homeostasis^136^. γδ T cells contribute to both innate and acquired immune responses during sepsis, with IFN-γ, IL-17, and other chemokines being released after γδ T cell activation.

In sepsis patients, γδ T cells are activated with increased surface expression of CD69 and HLA-DR, but the number of circulating γδ T cells is significantly lower than that of healthy subjects^137,138^, and the reductions correlate with the severity of illness and highest mortality rates^139,140^. This is probably because the apoptosis-induced loss of γδ T cells in the intestinal mucosa results in higher susceptibility to secondary infections as pathogens invade the circulation or peritoneal cavity (Fig. 3)^13^. The number of γδ T cells in the peripheral circulation decreases by as much as 80%^141^. Liao et al. found that changes in the function-related markers of γδ T cells in the blood of sepsis patients and impaired IFN-γ expression by γδ T cells after antigen stimulation are associated with mortality in sepsis patients^142^. Furthermore, γδ T cell deficiency impairs the immune defenses and increases the mortality risk in sepsis^143^. Expansion of the γδ T cell population increased the resistance of immunodeficient mice to bacterial infection^144^. Collectively, these findings show that preventing γδ T cell apoptosis could attenuate inflammatory responses^145^, providing new insight in the understanding of the functions of γδ T cells in sepsis.

### CD4^+^ T cells and associated subpopulations

T lymphocytes are key elements in all adaptive immune responses. CD4^+^ T cells are among the most important peripheral lymphocyte subsets in terms of modulating successful immune responses, influencing innate and adaptive immune cells through cytokine production, and cell-to-cell interaction^146,147^. When presented with peptide antigens, mature CD4^+^ T cells become activated and rapidly divide into several subsets that facilitate various immune responses by differing cytokine generation and secretion upon activation^148^. Among these cell subsets, Th1, Th2, and Th17 cells are major representatives of Th cells and are further explored here.

Apoptosis of T lymphocytes is critical in the pathophysiology of sepsis, and CD4^+^ T cells could directly mediate the host response to sepsis^149^. The number of T lymphocytes undergoing apoptosis is significantly reduced during sepsis and is even higher in non-survivors than survivors^150^. One of the most notable T cell defects induced by sepsis is the development of apoptosis, which destroys the CD4^+^ T cell population (Fig. 3)^151,152^. Uncontrolled apoptosis of CD4^+^ T cells causes marked lymphocytopenia, which is particularly serious because clonal expansions are critical to overcome potentially lethal infections. Patients who died of sepsis were found to have a much greater magnitude of CD4^+^ T cell apoptosis than survivors^23^.

Several treatments to prevent immunosuppression have targeted the apoptosis of CD4^+^ T cells in animal models^153,154^. Of note, IL-7 has emerged as a promising therapeutic agent because it has been found to be essential in preventing T cell depletion^155^. IL-7 administration has been shown to effectively improve T cell viability and trafficking and release of IFN-γ and restore the delayed-type hypersensitivity response to recall antigens^156^, which improves survival in sepsis. Another promising approach in reversing immunosuppression in sepsis involves blockade of the co-inhibitory molecules PD-1 and PD-L1. PD-1 blockade has been reported to increase the release of IFN-γ and prevent T cell apoptosis in patients with active infections^157^. However, the mechanisms inhibiting T cell apoptosis during sepsis are complex and yet to be completely understood^158^.

Not only the number of CD4^+^ T cell populations but also the function of the remaining lymphocytes is reduced during sepsis. Sepsis leads to uncontrolled apoptosis-induced depletion of CD4^+^ T cells, and some remaining cells are rendered dysfunctional or exhausted due to the prolonged exposure to excessive pro- and anti-inflammatory cytokines. CD4^+^ T cell exhaustion has been typified by T cells that have severely impaired effector functions and has been found in patients with sepsis. The prolonged duration of sepsis is characterized by high antigen load and elevated pro- and anti-inflammatory cytokines, which is conducive for T cell exhaustion^159^. The association between T cell exhaustion and increased mortality in sepsis is due to immune paralysis and secondary nosocomial infections^160,161^. Multiple independent studies have reported that blockade of the PD-1/PD-L1 pathway could attenuate T cell exhaustion, increase IFN-γ production, prevent apoptosis, and improve survival in various pathologic models of sepsis^162–164^. Collectively, these findings indicate that T cell exhaustion is also a major etiology of immune dysfunction in sepsis and that reversal of putative T cell exhaustion is a promising modality in the treatment of sepsis.

### B cells

B cells play an important role in both adaptive and innate immune response^165^. Under physiological circumstances, activated effector B cells could differentiate into plasma cells or memory B cells and drive the humoral immune response and act as APCs to activate effector T cells^166^. B cell function is relegated to the production of antibodies (Abs) and the development of memory plasma B cells^165^. Multiple studies have documented that impaired B cell function^167,168^, along with the production of IL-10^169,170^, the presentation of microorganism antigens to T lymphocytes^171^, and interaction of several bacterial products with B cells^165,172^, weakens immunity during sepsis. The relative proportion of B cell subsets is decreased on admission day in critically ill patients with sepsis, indicating that the apoptosis of B cells was induced^173,174^. In addition, endotoxemia causes a transient depletion of memory B cells and regulatory B cells from the circulation, but the functional capacity of B cells to produce IL-10 is maintained (Fig. 3)^175^.

Excessive apoptosis of B cells in sepsis likely involves multiple pathogen-sensing receptors and redundant signaling pathways. For example, Octavia et al. found that myeloid differentiation primary response gene 88 (MyD88) was effective in blocking the apoptosis of B cells, and MyD88 deficiency could markedly decrease B cells apoptosis, but the mortality rate significantly increased^176^. However, administration of Tubastatin A, a selective inhibitor of histone deacetylase 6, restored the percentage of B lymphocytes and significantly increased the percentages of innate immune cells and macrophages^177^. Therefore, therapies targeted at reversing B cell depletion should be actively investigated.

### Treg cells

Treg cells are a component of adaptive immunity that suppress responses of other effector T cell subsets, helping to maintain tolerance to self-antigens and suppression of autoimmune disease^178^. Treg cells are involved in the maintenance of peripheral tolerance and control of the immune processes^179,180^. Furthermore, Treg cells play a key role in controlling inflammation in many infectious diseases, including sepsis^180,181^. In sepsis and critical illness, Treg cells are detrimental to the proliferation and functional activity of effector T cells and other immune cells; for example, Treg cells inhibit both monocyte and neutrophil function^82^.

The number of circulating Treg cells increase in septic shock, particularly in the early stages after initiation. Further, although this increase was observed immediately after the onset of sepsis, it persisted only in those who subsequently died^182^. Further study revealed that the increase in Treg cells is caused by the loss of effector T cells rather than an absolute increase in Treg cells^183^. The involvement of these cells in long-term sepsis-induced immune dysfunction^184^ may be attributed to inhibition of monocyte function through a pro-apoptotic mechanism involving the Fas/FasL pathway^82^. Treg cells are less vulnerable to sepsis-induced apoptosis; therefore, the percentage of Treg cells increases in patients with sepsis^185,186^. Cell apoptosis is a continuous state, but Treg cells are more resistant to sepsis-induced apoptosis than other T cell subpopulations^1,187^, thus the increased ratio of Treg cells during the early period after sepsis. Increased Treg cell population would prevent recovery of the immune system from excessive immune responses. Importantly, these resistant effects are possibly due to TLR4 deficiency and associated NF-κB signal pathways^188^.

The higher number of Treg cells in sepsis patients impairs immunity and contributes to secondary infections and mortality by acting both on innate and adaptive immune cells (Fig. 3). However, the role of Treg cells in septic injury is yet to be clarified. Although prevention of Treg cell apoptosis inhibited immune responses or decreased survival in animal models of sepsis, the removal of Treg cells via anti-CD25 monoclonal Ab administration did not improve survival in animal model^189^. This may be because not only Treg cells but also other T cell subpopulations are depleted. In conclusion, Treg cells are more resistant to apoptosis in sepsis, thus potentially serving as targets for immune modulation.

## Clinical perspectives

Given the profound immunosuppression induced by depletion of immune cells that occurs during sepsis, the ability to sequentially follow the uncontrolled lymphocyte apoptosis as a means to evaluate the efficacy of immune-adjuvant therapies provides promising novel therapeutic opportunities^190^. The potential of preventing lymphocyte apoptosis as a treatment strategy in sepsis has been supported by many animal model studies. The first study to report that prevention of apoptosis improved the survival in sepsis showed that Bcl-2 overexpression is more resistant to sepsis-induced apoptosis^191^. Furthermore, an increasing number of immunoadjuvant therapies to prevent sepsis-induced immune paralysis have been identified as apoptosis dependent. IL-7 and anti-PD-L1 have been found to have potent effects to prevent lymphocyte apoptosis. IL-7 administration is an attractive therapy in sepsis, because it blocks sepsis-induced apoptosis of immune effector cells and increases IFN-γ, a cytokine that is critical for an effective host response against invading pathogens. This presents a potential novel strategy in the treatment of patients with sepsis by restoring adaptive immunity. Such immune-based therapy should be broadly protective against numerous bacterial and fungal pathogens^192^. More specifically, other agents, such as granulocyte colony-stimulating factor, granulocyte-macrophage colony-stimulating factor, IFN-γ, IL-15, anti-PD-1/PD-L1, and anti-B and T lymphocyte attenuator will target the immunosuppressed state in critically ill patients (Table 1)^193^. Therefore, development of a non-invasive imaging modality to detect and serially follow apoptotic cell death could be helpful in evaluating the efficacy of immunoadjuvant therapies in sepsis patients. This provides a bright prospect of autophagic modulation in clinical application.

**Table 1:** Immune modulators and proposed benefits for sepsis-induced apoptosis therapy

Immune modulator	Effects	Reference
G-CSF	Improve neutrophils and monocyte production and release	^194^
GM-CSF	Activate and induce production of neutrophils and monocytes or macrophages and reduce cell death	^195, 196^
IFN-γ	Increase monocyte expression of HLA-DR, increase numbers of IL-17 producing CD4^+^ T cells	^197, 198^
PD-1/PD-L1	Anti-apoptotic effects to prevent loss of protective function of NK cells Prevent lymphocyte apoptosis and reverse monocyte dysfunction	^132, 133, 199, 200^
IL-7	Blockade of sepsis-induced apoptosis depletion, increase production of CD4^+^ T and CD8 T cells Enhance trafficking of T cells to sites of infection	^1, 156, 201^
IL-15	Restrain sepsis-induced apoptosis of CD8 T cells, NK cells, and DCs	^130^
Tim-2-specific antibody	Decrease lymphocyte apoptosis and reverse the macrophage function	^202, 203^
Ulinastatin	Increase apoptotic rate of Treg cells and reduce the percentage through NF-κB pathway, ameliorate mortality	^188^
CTLA-4-specific antibody	Improve overall sepsis-induced lymphocyte apoptosis and survival of secondary fungal infections	^163, 204^

*G-CSF* granulocyte colony-stimulating factor, *GM-CSF* granulocyte-macrophage colony-stimulating factor, *IFN-γ* interferon gamma, *PD-1* programmed cell death-1, *IL* interleukin, *CTLA-4* cytotoxic T lymphocyte antigen-4

## Conclusion

Impaired apoptosis aggravates sepsis-induced immunosuppression in both innate and adaptive immune systems. Thus manipulating apoptosis could be a new therapeutic approach in sepsis. Further, exploring potential therapeutic targets related to apoptosis will be valuable in reversing sepsis-induced immunosuppression.

## Data Availability

The dataset used for this study is available from the corresponding author on reasonable request.
